# A Model to guide force-based manipulation research and practice

**DOI:** 10.1371/journal.pone.0331606

**Published:** 2025-09-12

**Authors:** M. Terry Loghmani, Damian Keter, Geoffrey M. Bove, Beth A. Winkelstein, Thomas C. Bulea, Håkan Olausson, Medha N. Pathak, Rachael Powell, Chad E. Cook

**Affiliations:** 1 Department of Physical Therapy, School of Health & Human Sciences, Indiana University, Indianapolis, Indiana, United States of America; 2 Physical Medicine and Rehabilitation Department, United States Department of Veterans Affairs, Cleveland, Ohio, United States of America; 3 Bove Consulting, Kennebunkport, Maine, United States of America; 4 Department of Bioengineering, School of Engineering and Applied Sciences, University of Pennsylvania, Philadelphia, Pennsylvania, United States of America; 5 Rehabilitation Medicine Department, National Institutes of Health Clinical Center, Bethesda, Maryland, United States of America; 6 Department of Biomedical and Clinical Sciences, Linköping University, Linköping, Sweden; 7 Department of Physiology & Biophysics, Sue and Bill Gross Stem Cell Research Center, University of California, Irvine, Irvine, California, United States of America; 8 Department of Orthopaedics, Duke University School of Medicine, Duke University, Durham, North Carolina, United States of America; 9 Department of Population Health Sciences, Duke University, Durham, North Carolina, United States of America; 10 Duke Clinical Research Institute, Duke University, Durham, North Carolina, United States of America; Erzurum Technical University: Erzurum Teknik Universitesi, TÜRKIYE

## Abstract

**Introduction:**

Manual therapies are forms of force-based manipulations (FBM) and involve the application of mechanical force to the outside of the body with therapeutic intent. The United States National Institutes of Health (NIH) U24 FBM Taxonomy and Terminology Committee (FBM-TTC) was formed to better understand why responses to FBM differ between individuals. One objective for this multi-disciplinary working group was to develop a framework outlining factors that should be considered, measured, and reported when developing and performing studies on FBM.

**Methods:**

The workgroup collaborated to develop a model outlining elements to consider during FBM research and practice. Three different models were proposed by members of the group who voted on a preferred model using a rank-ordered process and refined the selected model based on consensus and published literature.

**Results:**

A 3-dimensional (3D) matrix model was chosen that includes three elements: contextual factors influencing FBM outcomes, structure and function levels focusing on biological and physiological aspects, and force parameters. Each element expands into different components and sub-levels. The model is designed to be interactive, integrative, and dynamic.

**Discussion:**

The model provides a framework to guide protocol development for FBM mechanistic research and clinical outcome studies. For example, researchers can design more robust studies systematically varying force parameters by considering other matrix components, while clinicians may develop more personalized treatment plans. The model supports the complexity of mechanistic responses to FBM by integrating the multitude of intrinsic and extrinsic factors that impact responses. Detailed discussion of each element is beyond the scope of this paper; however, content experts are encouraged to expand on this dynamic model.

**Conclusions:**

An innovative 3D model was developed to guide FBM research. The framework integrates foundational elements and accommodates new insights, making it a valuable tool to advance FBM science and practice.

## Introduction

Force-based manipulations (FBMs) – also known as manual therapies – refer to the application of mechanical force to the outside of the body with therapeutic intent [1]. Examples include light or deep massage and touch, thrust and non-thrust manipulation, and even needling [2]. FBM is applied in a clinical environment by many types of healthcare practitioners and is studied by a variety of mechanistic and clinical researchers. Identifying the physiological mechanisms of FBM is critical to the advancement of the field, as well as to the clinical optimization of any therapeutic FBM intervention [3].

‘Physiological Mechanisms’ is a broad term used to describe the molecular, cellular, or whole systems processes through which therapeutic interventions exert beneficial effects on a disease or condition [4]. Currently, the mechanisms by which FBMs exert their effects are grossly understudied [2,3,5]. In preclinical research, FBM techniques exhibit both peripheral and central influences, which are theorized to modulate pain, increase mobility, and improve function [6]. Proposed mechanisms can be specific to the intervention provided (e.g., pain modulation) or shared with other treatments (e.g., improved relaxation), reflecting physiological and psychological interactions within the recipient [7,8].

Efforts to: 1) define and quantify contextual effects relevant to FBM; 2) better understand molecular, cellular, and circuit mechanisms that underlie the effects of various manual therapies; and 3) characterize and quantify the types of mechanical forces, has been the charge of a National Institutes of Health (NIH) commissioned interdisciplinary working group (hereby known as FBM-TTC: Force Based Manipulation – Taxonomy and Terminology Committee). The group is comprised of basic, translational, and clinician scientists with collective interests in FBM [9]. A primary objective of the FBM-TTC is to better understand why recipients of FBM can exhibit markedly different responses, a phenomenon that is consistent among all treatment applications [10], by representing the interactive, dynamic processes that occur during the application of FBM. To reflect these dynamic processes, the FBM-TTC focused on developing a model with three primary elements: a) contextual factors, b) structure and function levels, and c) force parameters. The process of selection was based on the literature and using an iterative process of group consensus. The purpose of the model is to support FBM research study design and grant proposal development with the goal of optimizing practice and advancing understanding in the field. The premise is that the model should be relevant across multiple disciplines and support inter-disciplinary FBM research and healthcare. The model aims to provide a framework for researchers, with supportive translational information that ties mechanistic findings to clinical outcomes.

## Methods

### Ethics statement

This committee project was commissioned by the NIH. Human subjects were not recruited. Participation on this project to recommend a model to help guide FBM research and practice was voluntary. The Indiana University Institutional Review Board determined this project is ‘Not Human Subjects Research’ as defined by federal regulations; thus, participant consent was not required.

### Participants

The multi-disciplinary FBM-TTC was formed by members from three National Institutes of Health (NIH) U24 Networks (ForceNET [11], NeuronS_MATTR [12], and SPINEWORK [13]). The FBM-TTC initiated their objective in March of 2023. Initially, eight FBM-TTC members met monthly to create a scaffolding for a taxonomical framework. In November of 2023, the FMB-TTC was expanded by five individuals to include scientists with backgrounds in affective touch, biophysics, mechanical forces shaping neural processes, bio-fabrication, and neuromuscular tissue engineering. The FBM-TTC consisted of scientists and clinicians, mostly from the United States of America (USA), with different professional backgrounds (Table 1).

**Table 1:** NIH FBM-TTC Working Group Member Profiles.

Committee Member Background	Expertise	Location	Publications Related to FBM	Years in FBM Research	Years in Clinical Practice	Years in Academic Teaching
Physical Therapist/ Clinical Scientist	Contextual factors influence on FBM	USA	10	2	11	2
Physical Therapist/ Clinical Scientist	Soft tissue manipulation effects on biological and clinical outcomes	USA	15	10	41	26
Physical Therapist/ Clinical Scientist	Musculoskeletal trials	USA	385	20	34	25
Chiropractor/ Biomedical Researcher	General practice	USA	60	34	35	30
Engineer/ Biomedical Research	Biomechanics, pain, neuroimmune regulation of pain	USA	140+	31	N/A	24
Clinical Neurophysiology and Neuroscience	Human somatosensory system	Sweden	120	37	33	29
Biophysicist	Cellular mechanotransduction	USA	27	14	N/A	8
Biomedical Engineer/ Biomechanist	Rehabilitation Robotics, Biomechanics	USA	47	12	12	N/A
Bioengineering	Cellular mechanobiology	USA	9	8	N/A	1

The clinical and/or research background, area(s) of expertise, location, number of publications involving mechanisms research in FBM, number of years in research, clinical practice, and teaching roles related to FBM for the NIH FBM-TTC working group members are summarized.

### Model construction

From March of 2023 to February of 2024 the FBM-TTC developed a summative model to guide FBM research and clinical practice. The group established criteria for the model, requiring that it must be interactive, integrative and translational, and dynamic in order to match the complexity of research in this arena and ultimately clinical practice. To remain responsive to an ever-evolving field, the model needed to be able to expand and accommodate new knowledge and understanding. The development of the model framework was based on review of published literature along with the clinical and scientific experience and consensus among the multi-disciplinary team of expert working group members.

## Results

### Framework proposals

Three potential frameworks were developed by members of FBM-TTC and presented to the working group. The first framework involved a three-dimensional (3D) matrix model that reflected a) contextual factors, b) structure and function levels, and c) force parameters. A second framework involved a sunburst plot model with nested rings that reflected layers of factors to consider and potential mechanisms within the outer layers of the model. A third framework was presented as a linear model, with an expected ‘response’ to the application of a FBM stimulus. The FBM-TTC members used Qualtrics to anonymously and confidentially rank order their preferred models. After a first round of voting, the third model (linear model) was rejected, and the group re-voted on models one and two. Model one was chosen with over 70% of the individuals voting on it as the first choice.

### Construction of the model

A sub-group of the FBM-TTC was formed to further develop the selected model to prepare for broad dissemination. An iterative process was used to revise the model based upon collective perspectives/experience and the published literature. For example, some components of the original model were expanded whereas others were eliminated or combined. A professional illustrator from Indiana University was recruited for graphic design of the model and its related concepts. Revisions were completed asynchronously and synchronously between the FBM-TTC sub-group members and with the working group, and eventually presented at the NIH U24 FBM Annual Meeting, June 2024, in Bethesda, Maryland, to gain feedback from all three networks.

### Feedback on the model

Feedback from all FBM-TTC members of the working group and other NIH U24 Networks was solicited from March 2024 to August 2024. This process of seeking voluntary feedback from members was accomplished through email and follow-up discussions by phone or virtually.

### Model overview

**Fig 1:**
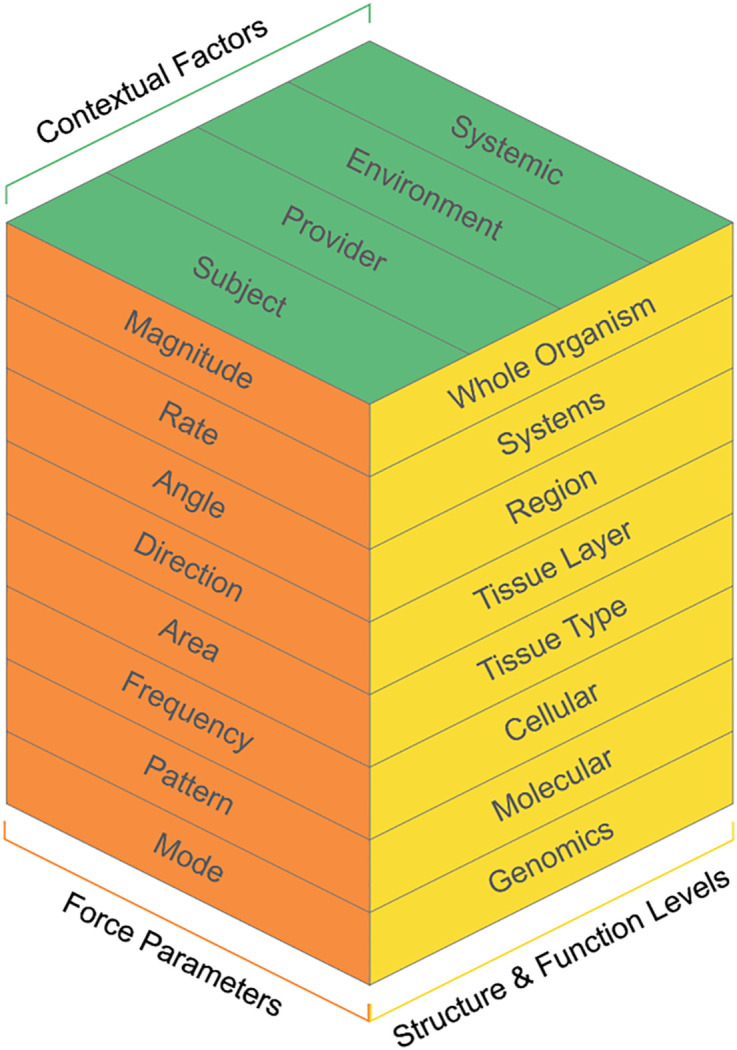
Model to Guide FBM Research and Practice.

A three-dimensional (3D) matrix model (hereby termed the FBM 3D model) was developed primarily to guide FBM research and to potentially assist in the clinical-decision making process for FBM interventions in practice (Fig 1). Three axes illustrate the elements of FBM and their relationships, including: a) contextual factors, b) structure and function levels, and c) force parameters. Each element is sub-divided into components and potentially sub-levels that should be considered for a comprehensive research plan and grant submission. An overview for each element, its components, and potential sub-levels is provided, and concept criteria discussed, below. The sequence of the components from top to bottom in each element does not necessarily represent the order of priority for consideration nor necessitate inclusion in a research proposal or therapeutic intervention plan. Quantification and measurement of component variables described in the model should be specified when able and as applicable, recognizing this can be discipline specific, dependent on the aims of a given study and resources, or lacking and in need of development. Readers are encouraged to refer to cited literature for more detail, since in-depth discussion of each component is beyond the scope of this manuscript.

### Description of model elements – contextual factors

“Contextual factors” refers to the intrinsic and extrinsic components that may moderate and mediate clinical decision making and the response to a given intervention [14]. These factors have been outlined as critical considerations in mechanistic research [8], and a consensus on gaps in knowledge in this area of study related to FBM has been recently established [2,5]. Contextual factors include subject factors, provider factors, systemic factors, and environmental factors, which may interact to influence mechanisms and clinical outcomes. These factors are briefly described and further summarized in Table 2.

**Table 2:** Examples of contextual factors demonstrating effect on FBM outcomes.

Subject Factors:		Supporting Literature:
Human:	Psychological Status (e.g., depression, anxiety, stress, fear, anxiety)	[15–21]
	Treatment expectations	[22–28]
	Physical Status (e.g., comorbid conditions, sleep status, fatigue, activity level)	[15,29–39]
Animal:	Fear and empathy Previous experience/expectations	[40,41]
*In vitro:*	Prior tissue use (previous load) Tissue storage conditions	[42–45]
**Provider Factors:**		
	Therapeutic alliance	[46–48]
	Rapport and empathy	[49,50]
	Physical attractiveness	[51]
**System Factors:**		
	Culture and religion	[52,53]
	Healthcare system	
	Payer	
**Environmental Factors:**		
	Lighting	[54]
	Noise/Music (auditory conditioning)	[55–57]
	Temperature	[58,59]
	Viewed experiences (visual conditioning)	[60]

#### Subject factors.

Human: Patient factors (e.g., psychological, nutritional, age, hormonal, and physical status) have the potential to act as moderators of clinical and mechanistic treatment outcomes [61]. These factors are variable and therefore should be described and measured as possible.

These factors are commonly based on validated patient reported measures to assess variables such as expectations [62], psychological status [63], and physical status [64]. A number of measures for each domain have been established therefore in study design researchers should select measures specific to their aims.

Animal (species: e.g., dog, rat, mouse): Animal models challenge the ability to measure self-reported subject factors, however, animals have shown the ability to develop fear, empathy, and preference for/against touch based on previous experiences and conditioning (for example, auditory, visual, and thermal). Previous experiences with the animal model should be described. Given inability to self-report these measures as in human models they are routinely captured by observation and behavioral analysis [65].

In vitro: Tissues studied *in vitro* are adaptive to mechanical load, therefore the source, previous use, and storage of the tissue should be considered contextual factors for *in vitro* research. As such, these factors should be tracked and described.

#### Provider factor.

Clinician and/or Researcher: Expectations and beliefs of the clinician and/or researcher and the context surrounding how the force is applied will influence response and should be described [49]. Measurement techniques are commonly self-developed to accommodate the research question and have successfully been used in FBM research design [66].

Other provider: Differences in force providers including self, robot (e.g., programmed to deliver precise load), or machine (e.g., roller table, massage chair) will influence the context surrounding force application and should be considered. These factors should be reported in as much detail as possible including use of photographs when able.

#### Environmental factors.

Location: The environment in which a force is applied will influence clinical and mechanistic outcomes. FBM may be delivered in clinical (e.g., hospital, hospice, outpatient, private practice, veterinary office), research (e.g., human, animal, cell labs) or home settings and should be described.

Sensory: Inputs influencing any of the senses (e.g., visual, auditory, olfactory, proprioceptive) should be described in detail for the subject (e.g., position, draping) and provider (e.g., position, equipment set-up).

#### Systemic factors.

Culture and Religion: The perception of and response to FBM may vary based on culture, religion, sex, and gender of the recipient (subject) and/or force applicator (provider) and should be considered.

Healthcare System: The process of healthcare delivery within a country/state/corporation on FBM healthcare and research, including what is available and perspectives on its value, need consideration.

Payer: Financial considerations impact the access and social equity of FBM-related healthcare and research. The availability or lack of resources for FBM can ultimately affect individual well-being (e.g., stress levels). The source of payment and/or reimbursement (e.g., out-of-pocket, insurance, workman’s compensation, litigation, state or federal government agencies and healthcare plans, foundations) should be considered.

### Description of model elements – structure & function levels

All FBM relies on the structure and function of both the person (or other entity) delivering the FBM and the subject receiving the FBM. Because of the high variability of form and constitution of both parties, the interaction is often unique. Structure and function should be considered on several levels during FBM research, including some or all those overviewed below, as applicable.

#### Whole organism.

The structure and function of the person/entity receiving FBM should be considered on a whole-body/organism level during FBM [67,68]. In human and animal models, pain, functional outcomes at home/work (school)/community (e.g., self-reported, observed), mobility and select clinical measures (e.g., physical performance measures, gait analysis), and indicators of overall, general health (e.g., cholesterol, mood indicators) may be considered. In cell models, motility and cytoskeletal structures need consideration, as do factors that would have affected the cells in their natural tissue’s habitat, including cell-to-cell mechanical or other communication.

Furthermore, while the provider carries strong contextual factors (see above), the physical makeup of the provider in terms of size and proportion dictates some force parameters and may drive the choice of technique. The FBM receiver’s habitus presents a broad array of challenges for therapists. Overall size is but one factor. Composition determined by muscle mass, fatty content, and activity level complicates matters for treatment delivery. The interface between the provider and the receiver is critical, as this influences multiple parameters of the FBM. Indeed, this interface may be the variable that is most difficult to understand, let alone define. Thus, the body position of the provider and receiver including the positioning of the limbs is critical to the impact of the FBM.

#### Systems.

The potential impact on body systems and their complex interactions should be routinely considered during FBM interventions in human and animal research models. Researchers should specify the system(s) under consideration. Mechanisms across different systems can be measured in a number of validated ways which have been described in a recent review [69]. Relevant systems include but are not limited to the following examples.

Neuromusculoskeletal and neurovisceral: The nervous system orchestrates the conscious and subconscious control of the entire body. FBMs are known to have direct and indirect impacts on this control, but since our understanding of the nervous system remains incomplete, our understanding of the mechanisms involving FBMs is limited. At the local or limb level, FBM may reduce the noxious input from a muscle or joint and has been proposed to help repattern normal neural function. Some evidence suggests local muscle twitch responses to needling applied to muscle trigger points may mediate relief of pain from musculoskeletal origin [70]. At the whole-body level, FBMs, especially to the axial structures, may have regulatory effects on visceral function. Somatovisceral reflexes may mediate such effects [71,72], but focal nerve inflammation secondary to musculoskeletal dysfunction has also been discussed as having a similar impact [73,74]. Thus, potential neurological mechanisms and pathways of interest should be specified (e.g., central, peripheral, sympathetic, parasympathetic, lymphatic, enteric, C-Tactile afferents system) [75].

Neuroendocrine: Touch has been shown to be critical to growth, oxytocin bonding, and pain modulation through neuroendocrine pathways [76]. Neuroendocrine pathways are also involved in regulating the stress response, and FBMs have been shown to have access to this physiology [77–79].

Autoimmune: Inflammation from autoimmunity underlies most rheumatic disease processes and a host of other syndromes, and the list is growing to include disorders such as fibromyalgia and types of neuropathic pain [80]. FBMs have been proposed to be helpful at least symptomatically in some related conditions; thus, exploring possible roles of FBM on autoimmunity, including neuroimmune cells, and cellular factors, warrants investigation and needs consideration [81,82].

#### Region.

Because of obvious anatomical differences, the region of the body treated (e.g., spine, upper, or lower extremity/quarter, etc.), or from which cells/tissue samples are harvested needs to be specified. The specific appendicular joint(s) (e.g., hip, interphalangeal, subtalar, etc.) and axial spinal joint/segments (e.g., intervertebral disc or facet, with level) need to be specified. If movement is being studied, the dynamics of the body part and/or joint(s) should be described. The anatomical position or relative relationship of the region of interest to other anatomical structure(s) should be described by using standard referencing nomenclature (e.g., anterior/posterior, medial/lateral, cephalad/caudal, sagittal, transverse, coronal) such that the treatment or study can be replicated.

#### Tissue layer.

In all regions of the body, structures are separated into layers by fascial planes. These are important because besides being highly innervated, the fascia and fascial interfaces allow movement that is required for normal function. The different depths of tissue layers (e.g., superficial, deep) potentially affected by FBM should be considered and indicated. The tissue layers under consideration or from which cell/tissue samples are harvested should be specified if relevant (e.g., epidermis/dermis, subcutaneous superficial fascia, deep fascia, muscular).

#### Tissue type.

Specific tissue types and/or structures under study or towards which FBM is directed should be indicated as precisely as possible, while acknowledging that the varied tissue types and structures that exist in a living organism involve complex interplays. A few examples of tissue types and structures are provided.

Nerve: The physical structural and function of nerves, spinal cord, and neurons may be studied in FBM research, e.g., conduction, axonal transport, and synaptic signaling. These structures are composed of different types of cells, including different subtypes of neurons, astrocytes, oligodendrocytes, as well as vascular and immune cells that support their functions.

Musculotendinous Unit: While massage therapy is philosophically focused on muscles and the tendons that arise from muscles, all FBMs have potential impact on muscles. Muscles and their tendons control most deliberate and reflexive movements, such as balance, all directed by the nervous system.

Smooth Muscle: Many FBMs are purportedly focused on visceral, lymphatic, and vascular structures. Direct and indirect impacts on the control of smooth muscles, including on the enteric nervous system, needs to be considered in all FBM clinical and research studies.

Bone: Most bones are somewhat flexible and are directly involved in some FBM and indirectly involved in most FBM. Cells that effect bone growth and metabolism are mechanically sensitive and may be meaningfully impacted by FBM [83].

Joint: FBM often affects joints and/or is directed toward joints. Joints allow for supported movements. Joints are under the control of muscles and extraneous forces, and are highly innervated, providing much information about position. They can be a source of pain that is out of proportion to their condition as evident by imaging or other examination. Tissues and structures associated with joints, e.g., cartilage, synovium, ligament, tendons under study during FBM research should be specified.

#### Cellular.

Mechanosensitive cells are potentially impacted by FBM via the processes of mechanotransduction and serve as cellular sub-levels under structure and function. FBM approaches should consider and specify potentially affected cell types affected or under consideration as able (e.g., fibroblasts/clasts/-cytes, osteoblast/clasts/-cytes, endothelial). The structure, such as intra- or extracellular architecture or organization, and/or function, such as signaling pathways, under consideration should be specified as relevant. Cells do not exist or function in isolation in living organisms, thus their complex interactions should be considered. Furthermore, the intricate relationship of cells living within an extracellular matrix should be considered. For example, FBMs have been shown to have modulating effects on inflammation, specifically through effects on fibroblasts [84]. Fibroblasts are highly mechanically sensitive [85], and FBM appears to affect their phenotype by preventing them from becoming unregulated myofibroblasts, which indiscriminately secrete collagen. Such collagen forms scar tissue and adhesions, which while necessary and desirable in some circumstances can become functionally problematic when left unchecked [86].

#### Molecular.

Very little is known about molecular effects of FBM. Yet, FBM has been shown to modulate TGF-beta and other inflammatory mediators that play key roles in the response to injury resolution [87]. Moreover, the signal transduction pathways by which the applied mechanical forces are transduced into biochemical signals within cells to modulate inflammatory mediators, repair factors, etc. need to be delineated.

#### Genomics.

Genomics may be considered a sub-level of molecular biology or an area of its own. The entire “-omics” field is open for exploration, including the genomic, proteomic, and metabolomic response to FBM approaches in healthy and disease conditions across the lifespan. It is possible that FBMs affect the function of genes that can be altered by mechanical stimuli, such as the cFos gene (in the pain pathway).

### Description of model elements – force parameters

The physics of FBM is highly complex, involving factors of the force provider and the subject or receiver of the force, and made more complicated by the points made in the above sections. Fundamentally, every force has both a magnitude and a direction, however several force parameters lack standardized and quantifiable measures in clinical practice and research. An intent of this model is to help address this issue while augmenting individualized patient care and allowing for diverse FBM approaches. It is beyond the scope of this model paper to provide an in-depth analysis of force parameters for these different approaches (e.g., massage, spinal manipulation) which have varying requirements and responses to force application but should nonetheless be considered. However, the integrative and dynamic nature of the model allow it to be catered to specific modes of FBM (e.g., massage, spinal manipulation) based on varying requirements and considerations. A potential impact could be that it helps catalyze efforts towards developing more standardized force measures.

When developing research approaches as well as delivering any treatment, several force parameters should be considered, described and/or documented. The overall consideration must be dose, or extent, of the treatment. Since treatments often consist of many components, the dose may need to include the sum of all the parameters of a particular treatment. This may also be considered as the amount of energy that is transferred from the provider to the receiver. In clinical practice, the provider may consciously choose the force parameters, but often the critical parameters contributing to a particular dose are subjective, may be somewhat subjectively or not determined a priori, and are guided by the situation as it develops, including instantaneous feedback from the subject and the tissue quality itself. In research, the parameters contributing to the dose must be carefully derived and described, while considering contextual factors, especially if the research is to be relevant to clinical practice. It is important to delineate the specifics of the treatment, including the force parameters described below.

#### Magnitude.

The magnitude is considered the strength of an applied FBM force. From a mechanical perspective, it is the amount of force. It is independent of direction but can include components from all three dimensions in space. While there is great specificity in engineering and physics-based communities around describing forces, force magnitude for clinical studies is often subjectively described (e.g., low, medium, or high force application). Force sensors/plates act as more precise objective measures which have been utilized to assess magnitude of FBM applications [88].

#### Rate.

Rate is a temporal variable. Simply, it is the speed (velocity) of the applied force. It can also be used to describe the speed of deformation – which is called strain rate. For many years, studies describing the mechanical responses and properties of biological structures, like ligaments and muscle, have shown them to be rate-dependent, with the speed of force application having varied effects on the response of the structure [89–91]. Thus, the speed of application, in some form, is an important factor and should be included in all descriptions of force. The term “rate” may also be used to refer to the number of repetitions applied over time, for example ‘stroke rate’, but actually applies to the parameter of ‘frequency’ described later.

#### Angle.

The angle of force application at the point of contact is known as the ‘line of action’ along which the force is intended to be distributed or act. It can be applied at any direction in space and not necessarily aligned with any particular anatomy. Forces that act along a primary coordinate axis of an object are referred to as axial. When forces are applied evenly across an object’s geometric center, it is called concentric, and when it is unevenly applied, it is called eccentric. The efficiency of force transfer can be negatively impacted if the angle of application is incongruent with the treatment intent. For example, the force applied at angles that are not at 90° to the surface (called “normal”) is lessened since there is only a fraction of the normal force transferred; however, if the desired effect is to target shear forces, the clinician may intentionally choose not to deploy a normal force.

#### Direction.

The direction of the force(s) being delivered is critical to the force transfer. Statically, the direction of a force may be described by the specific angles along a coordinate (x, y, z) axis system from a reference frame such as the body/entity surface. Dynamically, the direction of the force may refer to the motion path, or trajectory, of the force being applied relative to a reference point, such as any anatomical landmark.

#### Area.

The contact area and contour of the object delivering the force and the subject receiving the force need to be considered and described. If the object or subject are flexible or rate-dependent, contact area and contour will change during the force delivery, changing the force transfer and thus the dose. The effect of a contact force on tissue and force transfer depends on the area over which the force is applied, which for human tissue in contact with a more-rigid body can change based on geometry of the contacting object and amount of force. The magnitude of a compressive force divided by the area over which it is applied is called pressure.

#### Frequency.

Frequency can have many meanings in FBMs. Frequency may refer to the number of applied repetitions over time or be in relation to any oscillatory treatment. Frequency may also refer to the number of applications of a FBM intervention over time. For example, “dose-frequency” studies may explore the impact of how often a subject is treated per any defined time on outcomes, e.g., number of days/week, number of times/day. In these studies, the frequency may be considered to have individual or cumulative effects (effect per session or across sessions).

#### Pattern.

The pattern of forces may refer to the sequence in which individual forces are delivered. For instance, it is common to have lighter forces (like stroking) precede more intense forces (like focal deep pressure), thought to allow the organism to better adapt to the treatment.

#### Mode.

This refers to how a force is applied, such as manually by hand alone, with a rigid device (instrument-assisted) [92], or by using a massage-mimetic device [93]. Mode may also describe a “treatment approach” as performed by a group of practitioners. For example, cervical spinal mobilization is performed by many professions, such as massage therapy and chiropractic. However, the mobilization method, and resultant subjective experience, varies greatly by profession, and by individual clinicians, and should be indicated.

### FBM model features

#### Interactive.

**Fig 2:**
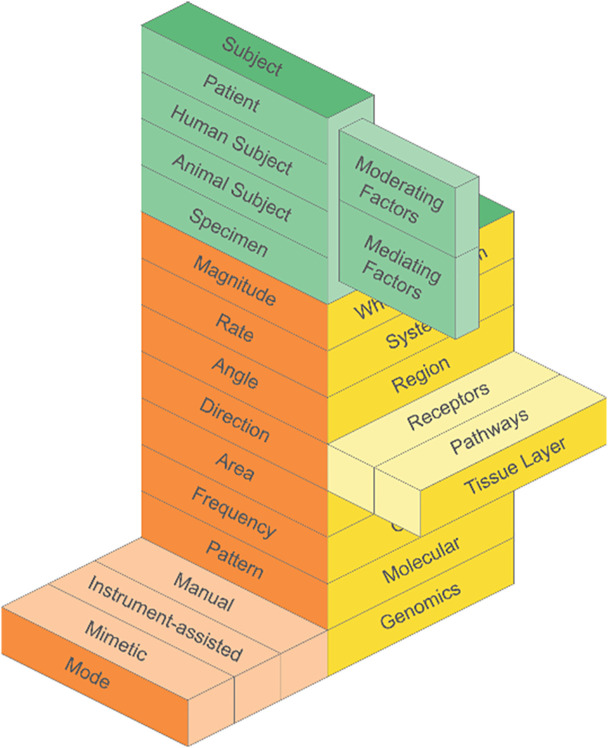
An Interactive Model. This model allows for broad or specific application of each of the components based on the needs of the researcher or clinician.

The model is designed to be interactive. Each component (“drawer”) can be “opened up” to deeper sub-levels and broader factors (Fig 2). For example, under “Contextual Factors” (green panel) the “Subject” component can be pulled up to consider relevant sub-levels, such as “Patient, “ “Human Subject,” “Animal Subject,” and “Specimen.” This “drawer” can be broadened to consider “Moderating Factors” and “Mediating Factors,” for example, as relevant to the subject. Fig 2 also depicts two other examples of interaction within the “Structure and Function Levels” (yellow panel) and “Force Parameters” (orange panel).

#### Integrative & translational.

**Fig 3:**
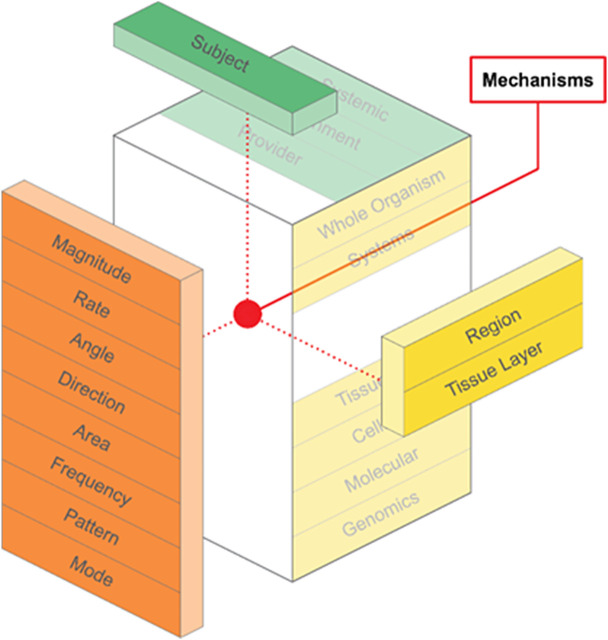
An Integrative Model. The model considers the intersection of the elements, components, and sub-levels for FBM mechanisms research.

**Fig 4:**
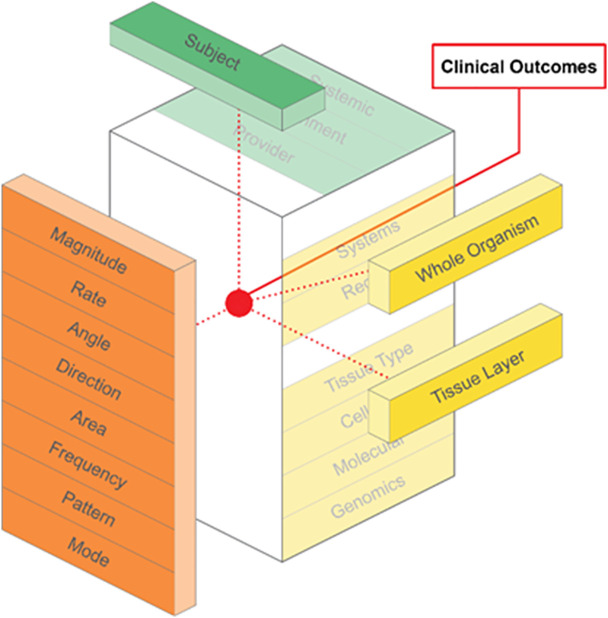
A Translational Model. The model can also be used to consider the intersection of the elements, components, and sub-levels for translational research on FBM approaches, including clinical outcomes.

The model is intended to integrate various components as required to explore the underlying mechanisms or clinical outcomes of FBM approaches (Fig 3 and 4). For example, all of the force parameters may be pre-determined when delivering soft tissue manipulation with a mimetic device to the quadriceps muscle layer in rodents with induced osteoarthritis of the knee to determine underlying mechanisms of FBM. As another example, components of the model should be considered, including description of the force parameters, during translational research exploring clinical outcomes on upper extremity pain and function in breast cancer patients with post-mastectomy scar tissue in response to a FBM approach.

#### Dynamic.

**Fig 5:**
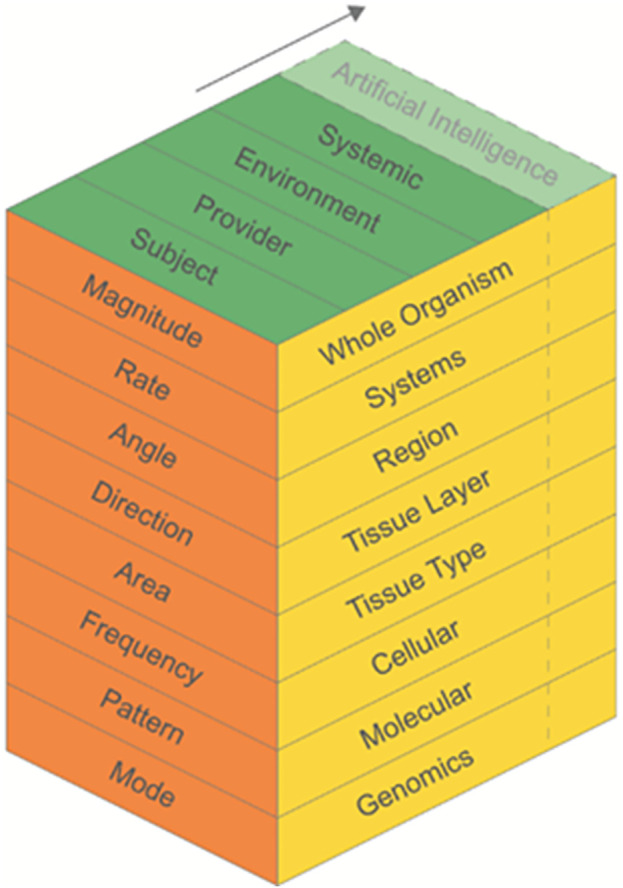
A Dynamic Model. The model is dynamic in that is allows components and sub-levels to be adapted, making it responsive to new understandings found in FBM research and practice.

The dynamic nature of the model is realized in several ways. First, each element can be expanded as necessary (Fig 5). Its modular structure enables the addition or modification of components and sub-levels as new information becomes available, ensuring the model can adapt to the ever-evolving FBM research environment. For instance, artificial intelligence (AI) might need to be added as a contextual factor due to its potential interaction with and influence on other components of the model. An example might be the use of AI during computational modeling of the impact of a FBM intervention on the response of different tissue layers or types (e.g., stiffness). The model also allows for adjustments and updates to be made, ensuring its continual refinement and accuracy. Furthermore, the model can be integrated dynamically with clinical practice, fostering its relevancy, practicality, and applicability.

The numerous components and sub-levels across the three axes highlight the complexity of FBM while also demonstrating the model’s flexibility and comprehensiveness. However, its implementation may initially seem overwhelming. For practical adoption, users are encouraged to selectively integrate the relevant components and sub-levels from their unique perspective. This approach should align specifically with the aims and objectives of a given project or patient, rather than attempting to address all aspects at once.

## Discussion

The FBM 3D model described in this manuscript is recommended by the NIH commissioned FBM-TTC working group to serve as an interactive guide in protocol development for use in mechanistic research and clinical studies involving FBMs. Previous frameworks have outlined methods to organize mechanistic response to FBM [6], force-based characteristics to consider when applying force to a specific tissue (skeletal muscle) [94] and terminology considerations to standardize FBM reporting [95]. Furthermore, previously developed models have attempted to summarize the multidimensional and dynamic nature of responses at a single structural level (fascia) [96]. To our knowledge the current model represents the first comprehensive model which 1.) accounts for all of the aforementioned considerations including appreciating non-specific factors such as contextual influence, 2.) can be applied to different types of FBM techniques, 3.) can be applied to animal, human, and in vitro studies, 4.) can investigate response at multiple different system and tissue levels based on the specific aims of the study.

Individuals with chronic health conditions such as persistent pain are increasingly turning to complementary and integrative health treatments such as FBM to enhance their well-being [97]. In fact, chronic pain is the leading indication for use of FBM interventions; about 33% of adults and 12% of children in the United States have reported the use of at least one FBM approach [97]. Despite its increasing use, FBM elements are poorly understood and lack a consistent structural framework that is shared across clinical and research disciplines. The FBM 3D matrix elements (contextual factors, structure and function levels, and force parameters) and their respective components and relevant sub-levels are important when describing research on FBM or during educational training of FBM providers.

The FBM 3D model has several important uses in research, including the development of research plans in the grant submission process. It may assist in designing FBM examination and treatment protocols; however, should not be considered prescriptive nor is it mandatory to include all of its components. With respect to education, the model may be useful in showing how modifications of FBM applications across the matrix components can allow adaptation of a conventional technique.

Ultimately, FBM research designed using the proposed model has immense potential to positively impact clinical practice by optimizing outcomes. Clinicians can use the model to tailor FBM treatments to patient-specific needs by adjusting force parameters based on the desired technique. Examples include the opening vs closing of a joint based on the angle and direction of force and joint manipulation vs mobilization based on the rate and magnitude of the thrust. Further work will be useful in identifying which measures of clinical outcomes each matrix component most notably influences.

The presented model outlines the importance of considering contextual factor influence when assessing mechanistic response to FBM. This model supports recent reviews which have shown that natural recovery without intervention, as well as non-specific effects associated with intervention influence treatment outcomes and may be more important in some cases than the force characteristics themselves [98,99]. The size of this effect however has demonstrated inconsistency with two recent reviews with meta analyses demonstrating significant difference in outcomes [99,100]. Factors outside of the specific force parameters themselves may account for the findings in several recent reviews which concluded that location of application and specifics of technique may be less important in influencing clinical outcomes than previous theorized [101,102].

Two recent reviews have established that if contextual factors are identified and targeted it may influence pain outcomes [103,104], however, the effect size within the included studies demonstrated significant heterogeneity questioning the value. Contextual factor influence on mechanistic outcomes is a complex field of study given its dynamic nature, the multitude of influencing factors, and the interaction between these factors themselves [8]. The individualized nature of factors influencing response to FBM support the response variability seen clinically with the application of FBM [105]. Clinicians should therefore appreciate the components of the presented model as it relates to clinical outcomes, including the individualized nature of clinical care and utilize person-centered care models to support this individuality [106].

The above breast cancer scar tissue scenario can be expanded on to better illustrate the guiding function of the model in designing studies. For example, if the aim is to determine the effects of massage on shoulder range of motion in this patient population, we could manipulate select force parameters (mode, intensity, duration, or frequency of massage), within consideration of contextual factors (patient’s health, activity levels, previous treatments), and prioritize assessment of structure/function levels on a whole-body level (pain assessment, range of motion measurements, functional tests). Whereas, if the aim is to explore the impact of massage on lymphedema, the focus of contextual factors may change (prioritizing patient’s lymphedema history, overall health) and structure and function levels could shift to the lymph system, involving tissue imaging (ultrasound, MRI), analysis of cellular and molecular inflammatory biomarkers (cytokines, chemokines). Pain and discomfort assessments, along with functional performance measurements (limb circumference, volume, mobility tests), could also be considered.

Despite its comprehensiveness, there are limitations to this model. First, it does not replace the judgement of basic scientists or clinicians in devising FBM studies or examination and intervention strategies. It is meant to augment the problem-solving and clinical decision-making inherent in the inquiry process and patient care. FBM intervention is based on an examination, evaluation, and plan of care, as appropriate, within the framework of established aims and objectives. Second, the model is meant to promote appreciation for the multitude of variables influencing the safety, effectiveness and efficiency of FBM interventions. Individual differences in the force perception of the provider and recipient of FBM interventions along with contextual factors and condition or structure being treated must be taken into consideration. The components of the FBM 3D model should be curated to the purpose and goals of the project or care. For example, if a patient being seen for nociceptive pain complaints after an injury presents with little to no negative contextual factors influencing their condition, focus may be on the force parameters and structure and function levels without significant focus on the contextual factors’ element. Third, given the model’s inherent complexity, the user should consider potential application barriers from a pragmatic standpoint, as they may present challenges for implementation, particularly in resource-limited settings or routine clinical use. Finally, the model is meant to accommodate new insights and approaches. Content experts are welcome to expand the model and contribute to building evidence for FBM approaches.

While this model presents a promising framework, its utility remains to be formally verified, necessitating further research to substantiate its efficacy and refine its application. To strengthen its scientific trajectory, future steps for empirical validation may include observational studies in educational settings, clinical case reports and controlled clinical trials, and tracking its use in mechanistic research and biomechanical assessments aimed to determine the therapeutic impact of FBM approaches. In the future, the FBM-TTC will collaborate with other FBM working group areas to evolve the model as new information arises. We see three potential mechanisms for continued updating. Firstly, the model was designed as a template for grant submissions, and we feel that FBM researchers will use and adapt pieces of our model. Second, we are making an animated and narrated video that will be available through different platforms (you tube; social media; personal and institutional websites) – that users/viewers can provide input (chat; discussion rooms). Lastly, we know of several other U24 mechanisms that address areas outside of FBM that are considering our model as a template. We feel their ongoing work can lead to modifications in our model as well.

The lack of conclusive evidence regarding FBM mechanisms and clinical effectiveness of FBM threatens the validity, reliability, and equitable availability of these non-invasive approaches. This FBM 3D model is relevant across multiple disciplines and will help guide basic research and clinical studies aimed to explore the mechanisms and outcomes of FBM as a conservative care option.

## Conclusions

An integrative and interactive FBM 3D matrix model is recommended to help guide FBM research, with potential implications in clinical practice. It was developed by members of the NIH commissioned FBM-TTC multi-disciplinary workgroup based on consensus and published literature. The model provides a foundation for elements that should be considered in developing grant proposals and research plans, including contextual factors, structure and function levels, and force parameters. The model can be expanded on by basic and clinician scientists from multiple disciplines as the field demands.

### Key points

1. An interactive, integrative, and dynamic 3-dimensional matrix model is recommended to help guide force-based manipulation (FBM) mechanisms research.
2. The purpose of this model is to support FBM research study design and grant proposal development, with the goal of improving safety, optimizing practice, and advancing understanding in the field.
3. The proposed model is adaptable, allowing elements and concepts to be expounded upon by content area experts, including in the areas of methodology and technology.
4. The model is designed for relevance across multiple disciplines and to support inter-disciplinary FBM research and healthcare.

## Supporting information

S1 Appendix Model Selection Data: Round 1. (CSV)

S2 Appendix Model Selection Data: Round 2. (CSV)
